# Obesity alters the mouse endometrial transcriptome in a cell context-dependent manner

**DOI:** 10.1186/s12958-022-01030-0

**Published:** 2022-11-24

**Authors:** Mike R. Wilson, Hilary Skalski, Jake J. Reske, Marc Wegener, Marie Adams, Galen Hostetter, Hanne M. Hoffmann, Jamie J. Bernard, Victoria L. Bae-Jump, Jose M. Teixeira, Ronald L. Chandler

**Affiliations:** 1 Department of Obstetrics, Gynecology and Reproductive Biology, College of Human Medicine, Michigan State University, Grand Rapids, MI 49503 USA; 2 Genomics Core Facility, Van Andel Research Institute, Grand Rapids, MI 49503 USA; 3 Pathology and Biorepository Core, Van Andel Research Institute, Grand Rapids, MI 49503 USA; 4 Reproductive and Developmental Sciences Program, Michigan State University, East Lansing, MI 48824 USA; 5 Department of Animal Science, Michigan State University, East Lansing, MI USA; 6 Department of Pharmacology and Toxicology, Michigan State University, East Lansing, MI 48824 USA; 7 Division of Dermatology, Department of Medicine, Michigan State University, East Lansing, MI USA; 8 Lineberger Comprehensive Cancer Center, University of North Carolina at Chapel Hill, Chapel Hill, NC 27599 USA; 9 Division of Gynecologic Oncology, University of North Carolina at Chapel Hill, Chapel Hill, NC 27599 USA; 10 Department of Epigenetics, Van Andel Research Institute, Grand Rapids, MI 49503 USA

**Keywords:** Uterus, Obesity, Endometrium, Epithelium, Stroma

## Abstract

Obesity impacts fertility and is positively correlated with endometrial hyperplasia and endometrial cancer occurrence. Endometrial epithelia often harbor disease driver-mutations, while endometrial stroma are highly regulative of neighboring epithelia. Here, we sought to determine distinct transcriptome changes occurring in individual cell types in the obese mouse uterus. Outbred CD-1 mice were fed high-fat or control diets for 18 weeks, estrous cycle staged, and endometrial epithelia, macrophages, and stroma isolated for transcriptomic analysis. High-fat diet mice displayed increased body mass and developed glucose intolerance, hyperinsulinemia, and fatty liver. Obese mouse epithelia displayed differential gene expression for genes related to innate immunity and leukocyte chemotaxis. The obese mouse stroma differentially expressed factors related to circadian rhythm, and expression of these genes correlated with glucose tolerance or body mass. We observed correlations between F4/80 + macrophage numbers, Cleaved Caspase 3 (CC3) apoptosis marker staining and glucose intolerance among obese mice, including a subgroup of obese mice with high CC3 + luminal epithelia. This subgroup displayed differential gene expression among all cell types, with pathways related to immune escape in epithelia and macrophages, while the stroma dysregulated pathways related to regulation of epithelia. These results suggest an important role for differential response of both the epithelia and stroma in their response to obesity, while macrophages are dysregulated in the context of apoptotic epithelia. The obesity-related gene expression programs in cells within the uterine microenvironment may influence the ability of the endometrium to function during pregnancy and influence disease pathogenesis.

**Supplementary Information:**

The online version contains supplementary material available at 10.1186/s12958-022-01030-0.

## Introduction

Obesity is defined by a disproportionate body weight relative to height and an accumulation of adipose tissue [1]. The global percentage of overweight or obese people has increased by 28% from 1980 to 2013 [2], and in 2014, the World Health Organization reported 1.5 billion overweight and 640 million obese people globally [1]. Obesity is associated with numerous chronic conditions, including type 2 diabetes, cardiovascular disease, and many types of cancer [1]. Obesity also has a negative impact on female fertility [3]. Evidence suggests that the uterine endometrium specifically is impacted by obesity [4]. Increases in BMI lead to reduced fecundity, even in eumenorrheic women [5–8]. Women with BMI > 30 displayed significantly lower rates of clinical pregnancy following frozen-thawed embryo transfer, which may indicate an effect of obesity on endometrial function [9].

In addition to its effects on fertility, obesity affects the pathogenesis of diseases derived from the endometrial epithelia, such as endometrial hyperplasia and endometrial cancer (EC) [10, 11]. EC was the first cancer identified with an increased risk among obese populations [10] and is the 4^th^ most commonly diagnosed cancer type in women [12]. Although EC mostly affects post-menopausal women, the incidence of EC is increasing at a higher rate among pre-menopausal women, likely due to increasing rates of obesity [13]. Conversely, endometriosis is a benign condition that is also thought to be derived from the endometrium and harbors many of the same mutations as EC, but incidence of endometriosis is thought to be negatively correlated with obesity [14]. Understanding the impact of obesity on the endometrium is imperative to developing preventative measures against EC and restoring fertility to obese women.

The endometrium is composed of a single cell layer of epithelial cells, which forms both luminal and glandular compartments, a multicellular highly regulated stromal layer, and additional vascular and immune components [15]. The endometrium is highly dynamic throughout the menstrual cycle, with changes in morphology occurring in response to ovarian steroid sex hormone levels. The endometrial stroma is thought to be most responsive to steroid hormone signaling, and stroma can, in turn, regulate the epithelia through paracrine interactions [16]. However, in most pathologies, the endometrial epithelium is the compartment that harbors genetic driver mutations, indicating the epithelia as the cell of origin for EC. Many classes of leukocytes reside within the endometrium and must be highly regulated during pregnancy to tolerate fetal antigens while also defending against infection [17]. The effect of obesity on uterine macrophages within the non-gravid uterus is relatively unexplored, although placental macrophages of obese women have been shown to have a pro-inflammatory phenotype [18]. An examination of the direct impact of obesity on the varying cell types of the endometrium will shed light onto the dysregulation of each cell type and their individual contributions to infertility and cancer pathogenesis.

In this study we sought to characterize the effects of obesity on the cell types in the endometrium. We utilized a mouse model of high fat diet-induced obesity [19] in combination with established methods for isolating mouse endometrial epithelia [20], newly developed methods to isolate the endometrial stroma and macrophages and RNA-seq analysis to identify obesity-associated transcriptomes within each cell type. The results described herein increase our understanding of the impact of obesity on the endometrium.

## Methods

### Mice

Female CD-1 mice (purchased from Charles River Laboratories) were provided with control diet (Bio-Serv, Cat# F4031) or high-fat diet (Bio-Serv, Cat# F3282) ad libitum for 18 weeks beginning at 8 weeks of age. Mice were monitored for signs of severe illness, such as dehydration, hunching, jaundice, ruffled fur, signs of infection, or non-responsiveness. Mice were kept on a 24-h light cycle, with light phase from 7 AM (Zeitgeber time; ZT0) to 7 PM (ZT12), and dark from 7 PM (ZT12) to 7 AM (ZT0). Mice and food were weighed weekly at ZT2 using a portable laboratory scale. Mouse blood samples (~ 0.2 mL) were collected every other week at ZT2 from the submandibular vein using a goldenrod lancet (cheek punch). Mice were sacrificed after 18 weeks on high-fat diet by CO_2_ inhalation. At time of sacrifice, estrous stage was identified by vaginal cytology [21]. Mice were housed at the Michigan State University Grand Rapids Research Center in accordance with protocols approved by Michigan State University, Michigan State University Institutional Animal Care and Use Committee (IACUC) protocol #202,200,067. Michigan State University is registered with the U.S. Department of Agriculture (USDA) and has an approved Animal Welfare Assurance from the NIH Office of Laboratory Animal Welfare (OLAW). MSU is accredited by the Association for Assessment and Accreditation of Laboratory Animal Care (AAALAC).

### Glucose tolerance test

Prior to performing the glucose tolerance, mice were fasted for 6 h (beginning at ZT2). Mice were weighed, and the tip of the tail was cut with sterile scissors and massaged until a drop of blood was obtained for time = 0 min measurement (at ZT8). Glucose levels were measured using a glucometer (One Touch Ultra 2 Blood Glucose Monitoring System) and test strips (True Point Generic Test Strips). Then, mice were injected interperitoneally with 2 g/kg of autoclaved USP dextrose (EMD Millipore, cat# G8270) dissolved in 0.9% saline (0.2 g/mL dextrose). Glucose levels were measured at 30, 60, 90, 120 and 150 min by drawing blood from the tail by removing the scab and massaging the tail until a drop of blood was obtained. Mice were tested for glucose tolerance after 17 weeks on control diet or high-fat diet (25 weeks of age).

### Insulin ELISA

Mouse serum was tested for insulin using the Mouse Insulin ELISA (Mercodia, cat# 10–1247-01) as described in the manufacturer’s instructions. Briefly, 10 µL of serum or calibrator was added to the wells, 100 µL of enzyme conjugate solution was added, and the plate was incubated for 2 h at room temperature at 700 rpm on a plate shaker. Each well was washed 6 times with 700 µL of wash buffer, and 200 µL of Substrate TMB was added to each well followed by a 15-min incubation at room temperature. Then, 50 µL of Stop Solution was added to each well, the plate was shaken for 5 s and then absorbance was measured at 450 nm using a SpectraMax i3x plate reader (Molecular Devices). Insulin concentrations were calculated based on calibrators provided in the kit.

### Cell sorting and purity analysis

Mouse uterine cells were isolated using modifications to our previously described method for isolating mouse endometrial epithelia [20]. After 18 weeks, mice were sacrificed at ZT2, and uteri were surgically removed and minced using scissors. Tissues were digested using the MACS Multi Tissue Dissociation Kit II (Miltenyi Biotec, cat# 130–110-203) for 75 min at 37° C. Digested tissues were strained through a 40 μm nylon mesh (Fisher Scientific, cat# 22–363-547). The Red Cell Lysis Buffer (Miltenyi Biotec, cat# 130–094-183) and the MACS Dead Cell Removal Kit (Miltenyi Biotec, cat# 130–090-101) were used to remove red blood cells and dead cells, respectively. For cohort 1 mice (5 control diet mice and 5 high-fat diet mice), epithelia were isolated as previously described. For cohort 2 mice (4 control diet mice and 8 high-fat diet mice), epithelia, macrophages and stroma were isolated from each animal. Macrophages were isolated using anti-F4/80-labeled MicroBeads (Miltenyi Biotec, cat# 130–110-443). Epithelia were positively selected and purified using a PE-conjugated EPCAM antibody (Miltenyi Biotec, cat# 130–117-753) and anti-PE MicroBeads (Miltenyi Biotec, cat# 130–048-801). Following isolation of macrophages and epithelia, the flow-through cells were collected as the endometrial stroma. A BD Accuri C6 flow cytometer (BD Biosciences) was used to confirm purity of macrophages using a PE-labeled F4/80 antibody (Miltenyi Biotec, cat# 130–116-499). Epithelial purity was confirmed using a PE- conjugated EPCAM antibody (Miltenyi Biotec, cat# 130–117-753). To confirm stroma purity, cells were fixed with 4% formaldehyde at room temperature. Cells were washed with PBS, permeabilized with ice-cold 100% methanol for 15 min, washed with PBS and incubated with 4 µg/mL of PE-labeled anti-Vimentin (Cell Signaling, cat# 12,020) or PE-labeled rabbit IgG (Cell Signaling, cat# 5742) for one hour at room temperature. Purity of fixed cells were confirmed by flow cytometry. Epithelia were collected from 21 mice, while macrophage and stroma were collected from 11 mice, at the endpoint of 18 weeks.

### RNA isolation, library preparation and RNA-sequencing

The Arcturus PicoPure RNA Isolation Kit (ThermoFisher, cat# 12,204–01) and the RNAse-free DNAse set (Qiagen, cat# 79,254) were used for RNA purification and on-column DNA digestion, respectively, using extracts from purified cells (*n* = 21 epithelia samples from cohorts 1 and 2, *n* = 11 stroma samples from cohort 2, *n* = 11 macrophage samples from cohort 2). RNA concentration and quality was assayed using a High Sensitivity DNA Chip on a Bioanalyzer 2100 (Agilent Technologies, Inc.). RNA samples had a mean RNA Integrity Number of 8.9 ± 0.8. Libraries were prepared by the Van Andel Genomics Core from a minimum of 180 pg of total RNA using the SMART-Seq v4 Ultra Low Input RNA Kit for Sequencing, v. 091,817 (Takara Bio USA). The Nextera DNA flex library prep kit (Illumina) was used to generate Illumina compatible sequencing libraries. In short, output of the cDNA reaction was introduced into the Nextera transposase reaction per protocol recommendations, after which index and adapter sequences were added through PCR amplification. Quality and quantity of the finished libraries were assessed using Agilent DNA High Sensitivity chip (Agilent Technologies, Inc.) and QuantiFluor® dsDNA System (Promega Corp.). Individually indexed libraries were pooled and 50 bp, paired end sequencing was performed on an Illumina NovaSeq6000 sequencer using an S1, 100 cycle sequencing kit (Illumina Inc.) and each library was sequenced to an average raw depth of 25 M reads. Base calling was done by Illumina RTA3 and output of NCS was demultiplexed and converted to FastQ format with Illumina Bcl2fastq v1.9.0.

### RNA-seq analysis

Generated, raw, 50 bp paired-end reads were trimmed via *cutadapt* [22] and *Trim Galore!* (http://www.bioinformatics.babraham.ac.uk/projects/trim_galore/). Quality control analysis was performed using *FastQC* [23] and *MultiQC* [24]. Trimmed reads were aligned to mm10 genome assembly, indexed to GENCODE [25] (vM16) GFF3 annotation via *STAR* [26] aligner with flag ‘–quantMode GeneCounts’ for feature counting. Reverse-stranded, gene-level counts were extracted from the STAR output files and constructed into an experimental read count matrix in R. Low count genes were filtered (1 count per sample on average) prior to *DESeq2* [27, 28] count normalization and subsequent differential-expression analysis. Calculated differential-expression probabilities were corrected for multiple testing by independent hypothesis weighting [29] for downstream analysis. Principal component analysis was calculated using *DESeq2* from top 500 genes by variance across samples. Heatmaps were generated using scaled regularized-logarithm counts (*DEseq2* function ‘rlog’) for visualization. Significantly differentially expressed genes were defined as *FDR* < 0.05. *DESeq2* output data is presented in Supplementary Data 1. For comparisons to mutant endometrial models, previously published RNA-seq data from *LtfCre*^*0/*+^; (Gt)R26Pik3ca^*H1047R^*; Arid1a*^*fl/fl*^ mice [20], *LtfCre*^*0/*+^*; Brg1*^*fl/fl*^ mice [30], and *LtfCre*^*0/*+^; (Gt)R26Pik3ca^*H1047R^*; Trp53*^*fl/fl*^ mice [31] were extracted from GEO accession series GSE129784, GSE152663 and GSE184499, respectively.

### Histology and immunohistochemistry

For indirect immunohistochemistry (IHC), 10% neutral-buffered formalin-fixed paraffin sections were processed for heat-based antigen unmasking in 10 mM sodium citrate (pH 6.0), Sections were incubated with primary antibodies at the following concentrations: 220 ng/mL Cleaved Caspase-3 (Cell Signaling, cat# 9579, RRID: AB_10897512); 675 ng/mL Ki67 (Cell Signaling, cat# 12,202, RRID: AB_2620142); 200 ng/mL KRT8 (Developmental Studies Hybridoma Bank, cat# TROMA-I, RRID:AB_531826). Biotin-conjugated secondary antibodies were used at the following concentrations: 4.8 µg/mL donkey anti-rabbit IgG (Jackson Immuno-Research Lab, cat# 711–065-152, RRID: AB_2340593) and 1.5 µg/mL donkey anti-rat IgG (Jackson Immuno-Research Lab, cat# 712–065-153, RRID: AB_2315779) were used as secondary antibodies and detected using VECTASTAIN Elite ABC HRP Kit (Vector Labs, cat# PK-6100). For negative controls, tissue sections were treated with both donkey-anti rabbit IgG and donkey anti-rat IgG secondary antibodies without a primary antibody. Sections for IHC were lightly counter-stained with Hematoxylin QS (Vector Labs, cat# H-3404–100). Routine Hematoxylin and Eosin (H&E) staining of sections was performed using Gill’s Hematoxylin #3 (Polysciences, cat# 24,244–500), Scott’s Bluing Reagent (Polysciences, cat# 24,605–1) and Eosin Y (Polysciences, cat# 09,859). Every mouse was assayed for each immunohistochemical marker and for negative controls.

### Bioinformatics and statistics

Various *ClusterProfiler* [32] functions were used to calculate and visualize pathway enrichment from a list of gene symbols or with respective gene universes. *biomaRt* [33, 34] was used for all gene nomenclature and ortholog conversions. *ggplot2* [35] was utilized for plotting applications*. ComplexHeatmap* [36] was utilized for hierarchical clustering by Euclidean distance and visualization. *eulerr* was utilized to produce proportional Euler diagrams [37]. The cumulative hypergeometric distribution was utilized for enrichment tests. Broad GSEA [38] was performed via GenePattern [39] on ortholog-converted *DESeq2* normalized counts from experimental mouse data. The statistical computing language R [40] and GraphPad Prism 9 software were used for many applications throughout this manuscript.

## Results

### High-fat diet in outbred CD-1 mouse model results in overeating and increased body mass

**Fig. 1:**
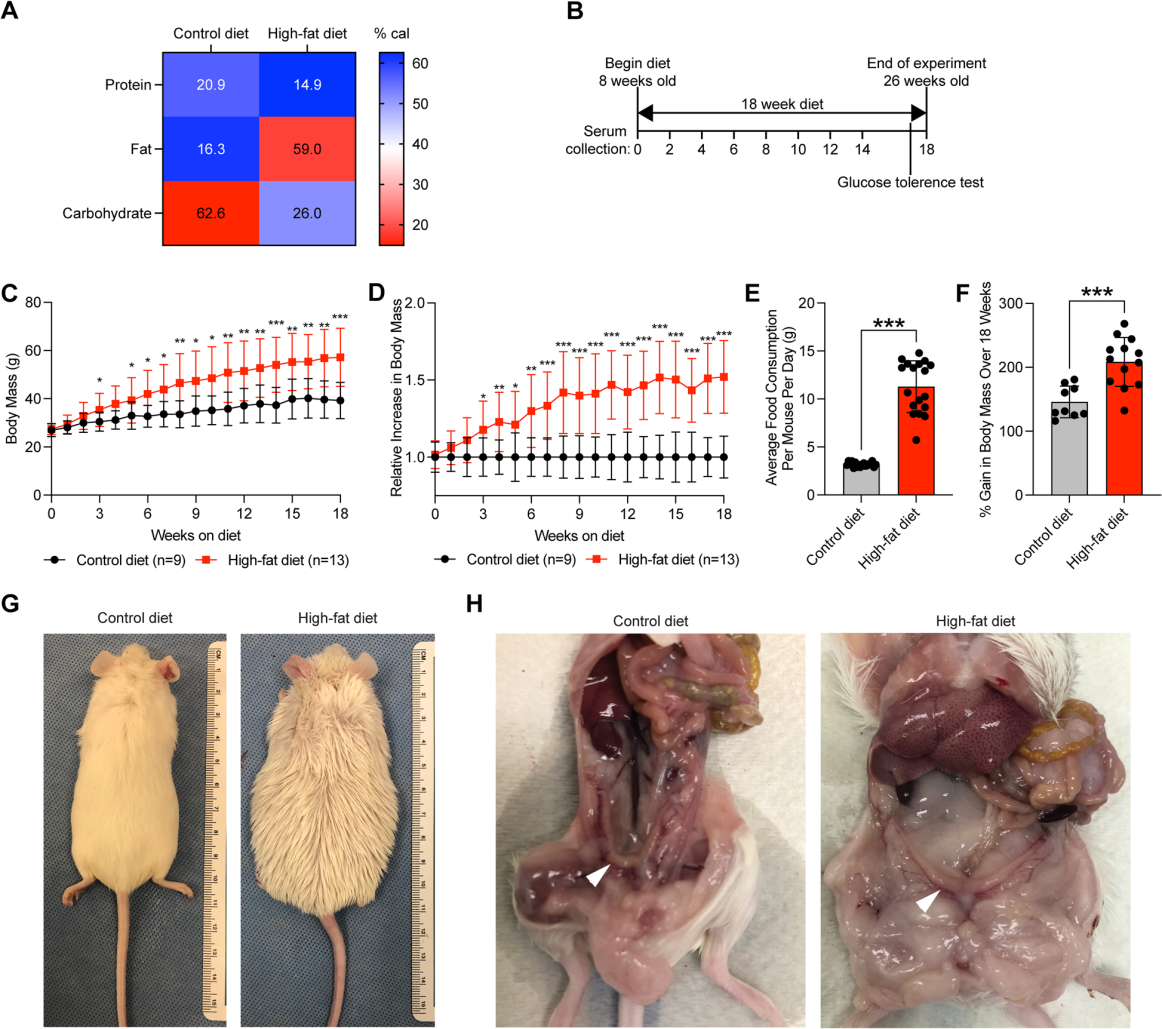
High-fat diet results in body mass gain in CD-1 mice. **A** Heatmap representation of caloric contribution of protein, fat and carbohydrate for control diet and high-fat diet. **B** Experimental timeline. **C** Body mass of female mice on control diet (*n* = 9) and high-fat diet (*n* = 13) mice (grams) over time, beginning at week 0 of diet. Measurements represent mean ± standard deviation (S.D.). Statistic is two-tailed, unpaired t-test between two groups at same timepoint. **D** Relative body mass of mice on high-fat diet (*n* = 13) relative to mice on control diet (*n* = 9) within the same cohort, mean ± S.D. Statistic is two-tailed, unpaired t-test between two groups at same timepoint. **E** Average food consumption per day per mouse (grams), mean ± S.D. Measurements were made weekly. Statistic is two-tailed, paired t-test. **F** Gain in body mass after 18 weeks for each mouse relative to body mass at week 0 for control diet (*n* = 9) and high-fat diet (*n* = 13), mean ± S.D. Statistic is unpaired t-test. **G, H** Gross images of control diet and high-fat diet mice after 18 weeks on diet. Uterus is denoted with arrowhead in panel H. *p < 0.05; **p < 0.01; ***p < 0.001

In order to observe the phenotypic effects of obesity in a model that mimics the genetic diversity of human populations [41], we utilized the CD-1 outbred mouse model [19]. Obesity was induced in CD-1 mice using a high-fat diet, in which 59% of calories came from fat, compared to control diet in which most calories came from carbohydrates (Fig. 1A). Two cohorts of female CD-1 mice were placed on a high-fat diet or control diet ad libitum beginning at 8 weeks of age (Fig. 1B). After just three weeks, mice on the high-fat diet displayed significant increases in body mass compared with mice on the control diet (Fig. 1C). The difference in body mass between high-fat diet mice and control diet mice continued to grow over the duration of the experiment, with an average 152% increase over the average body mass for high-fat diet mice compared to control mice (Fig. 1D). High-fat diet mice consistently ate an increased amount of food in total grams compared with control diet mice (Fig. 1E). Relative to body mass at week 0, the high-fat diet mice increased in mass by 209%, while the control diet mice increased in body mass by only 146% (Fig. 1F). After 18 weeks, the difference in body mass was apparent by eye (Fig. 1G). Upon gross inspection of mice after 18 weeks of diet, the uteri of high-fat diet mice appeared visually normal, while mice also harbored apparent features of obesity including increased adipose tissue and hepatic steatosis (Fig. 1H).

### Obese mice are glucose intolerant and hyperinsulinemic

**Fig. 2:**
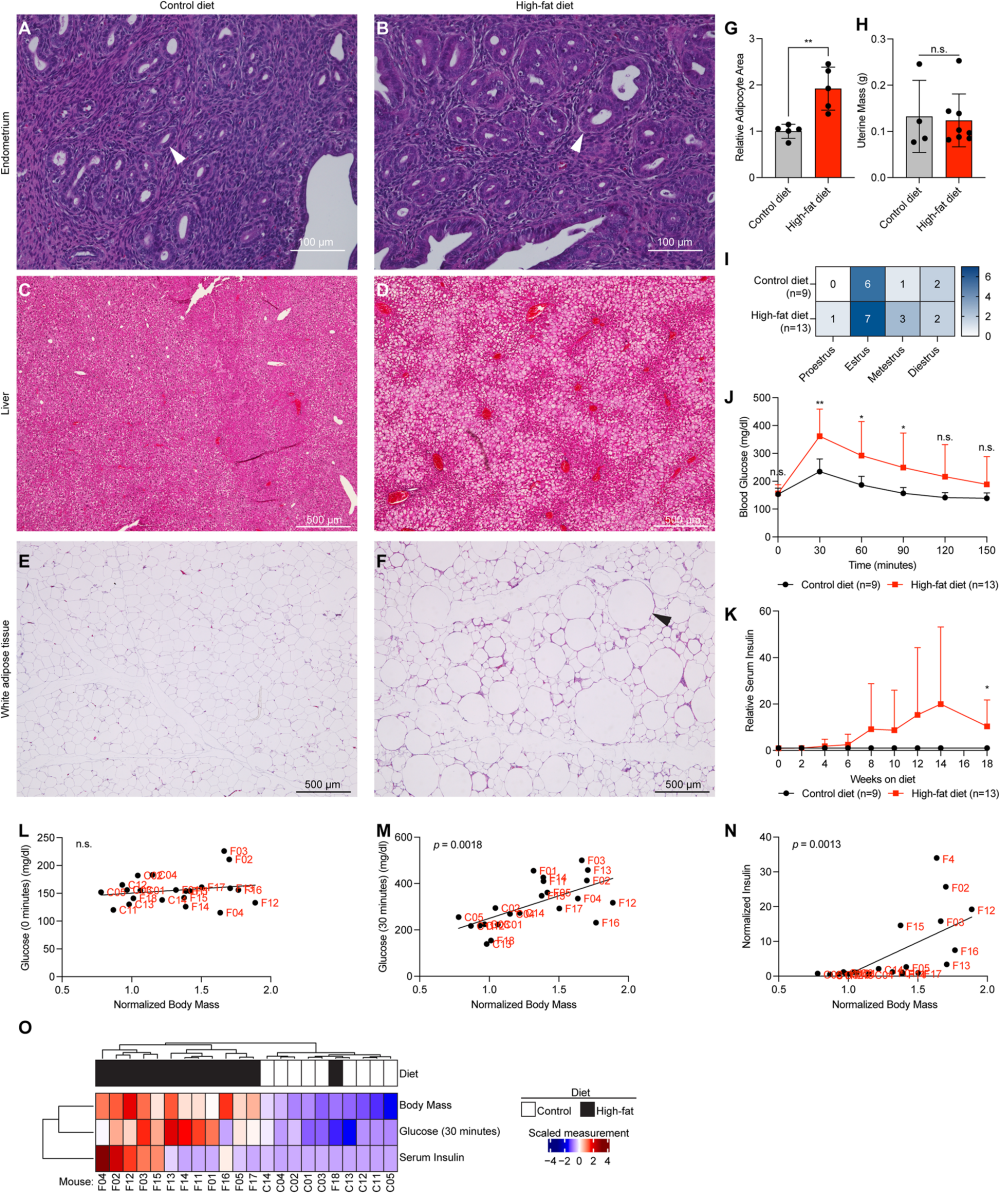
High-fat diet induces obese phenotypes in CD-1 mice. **A**,**B** Representative H&E staining for control and high-fat diet mouse uterus. **C**,**D** Representative H&E staining for control and high-fat diet mouse liver. The high-fat diet animals show accumulation of ectopic fat. **E**,**F** Representative H&E staining for control and high-fat diet mouse white adipose tissue. Arrowhead indicates hypertrophic adipocyte. **G** Quantification of area of adipocytes from histological sections in control diet (*n* = 5) and high-fat diet (*n* = 5), mean ± S.D. Statistic is two-tailed, unpaired t-test. **H** Semi-dry uterus mass measurement for control diet (*n* = 4) and high-fat diet (*n* = 8) mice, mean ± S.D. Statistic is two-tailed, unpaired t-test. **I** Heatmap representation of estrous cycle staging by vaginal swab test at time of uteri collection for control diet (*n* = 9) and high-fat diet (*n* = 13). Control diet and high-fat diet groups were not significantly different (Chi-square). **J** Blood glucose measurements over time in mg/dl after injection with 2 g/kg glucose for control diet (*n* = 9) and high-fat diet (*n* = 13), mean ± S.D. Statistic is two-tailed, unpaired t-test between two groups at same timepoint. Test was performed at week 17 of diet. **K** Measurements of serum insulin over time for control diet (*n* = 9) and high-fat diet (*n* = 13) mice determined by ELISA, normalized to cohort controls, mean ± S.D. Statistic is two-tailed, unpaired t-test. **L** Body mass of each mouse at week 18 normalized to cohort control (x-axis) vs. resting glucose measurement (t = 0, glucose tolerance test, mg/dl) (y-axis). Statistic is Pearson’s correlation. **M** Body mass of each mouse at week 18 of diet normalized to cohort control (x-axis) vs. glucose tolerance (t = 30, glucose tolerance test, mg/dl) (y-axis). Statistic is Pearson’s correlation. **N** Body mass of each mouse at week 18 of diet normalized to cohort control (x-axis) vs. serum insulin of each mouse at latest timepoint normalized to cohort control (y-axis). Statistic is Pearson’s correlation. **O** Hierarchical clustering of mice based on glucose (30 min), body mass and serum insulin measurements. *p < 0.05; **p < 0.01; ***p < 0.001; n.s. is non-significant

Tissues were collected, formalin-fixed and paraffin-embedded for histological sectioning followed by hematoxylin and eosin staining. No obvious phenotypic changes occurring in the endometrium were observed following 18 weeks on high fat diet (Fig. 2A-B). Obese mice appeared to have signs of fatty liver disease, with increased ectopic fat located in the liver (Fig. 2C-D). Additionally, the white adipose tissue in obese mice was hypertrophic, with the relative size of adipocytes increased in high-fat diet mice (Fig. 2E-G). At the endpoint of the experiment, the weight of the uteri (Fig. 2H) and estrous cycling (Fig. 2I) were not significantly different between the groups.

Since obesity contributes to impaired glucose metabolism, we tested glucose tolerance in high-fat diet mice and control diet mice after 17 weeks on their respective diets. We observed that high-fat diet mice had impaired glucose tolerance, with significantly increased blood glucose at 30-, 60- and 90-min following glucose injection (Fig. 2J). Obesity can also result in hyperinsulinemia, so we examined the insulin levels of mouse serum during the time-course of the experiment. Serum insulin levels were increased in mice treated with high-fat diet at 18 weeks (Fig. 2K). Resting glucose was not correlated with body mass (Fig. 2L), while glucose intolerance and serum insulin levels both displayed a significant linear correlation (Fig. 2M-N). Based on glucose tolerance, body mass and insulin levels, all mice were grouped together following hierarchical clustering, except for one high-fat diet mouse (Fig. 2O). This outlier mouse was excluded from further analysis. Overall, these results show that high-fat diet induces obese phenotypes in CD-1 mice, including glucose intolerance, hyperinsulinemia, fatty liver, and hypertrophic adipose tissue.

### The identification of novel cell identity markers from sorted uterine cells

We next aimed to characterize the transcriptomic changes occurring in several endometrial cell types during obesity. In addition to the two major cell types of the endometrium, the epithelia and the stroma, we isolated uterine macrophages, having observed an increase in the abundance of macrophages in the high-fat diet mouse endometrium (Supplemental Fig. 1A-E). Using magnetic sorting methods, we isolated epithelial cells, stromal cells, and macrophages from uteri of control diet mice and high-fat diet mice after 18 weeks on the diets (Supplemental Fig. 2A). Purity of the cell populations was confirmed by flow cytometry (Supplemental Fig. 2B-G). We observed differences in cell number between cell types, with the highest cell number from the epithelia (Supplemental Fig. 2H) However, the purity and cell number were not significantly different between control and high-fat diet uteri within the same cell type (Supplemental Fig. 2I-J). Epithelia cell purity was comparable to previous studies [20]. RNA was collected from purified cell populations, and by RNA-seq we detected 26,145 total expressed genes among epithelia, macrophages, and stroma.

**Fig. 3:**
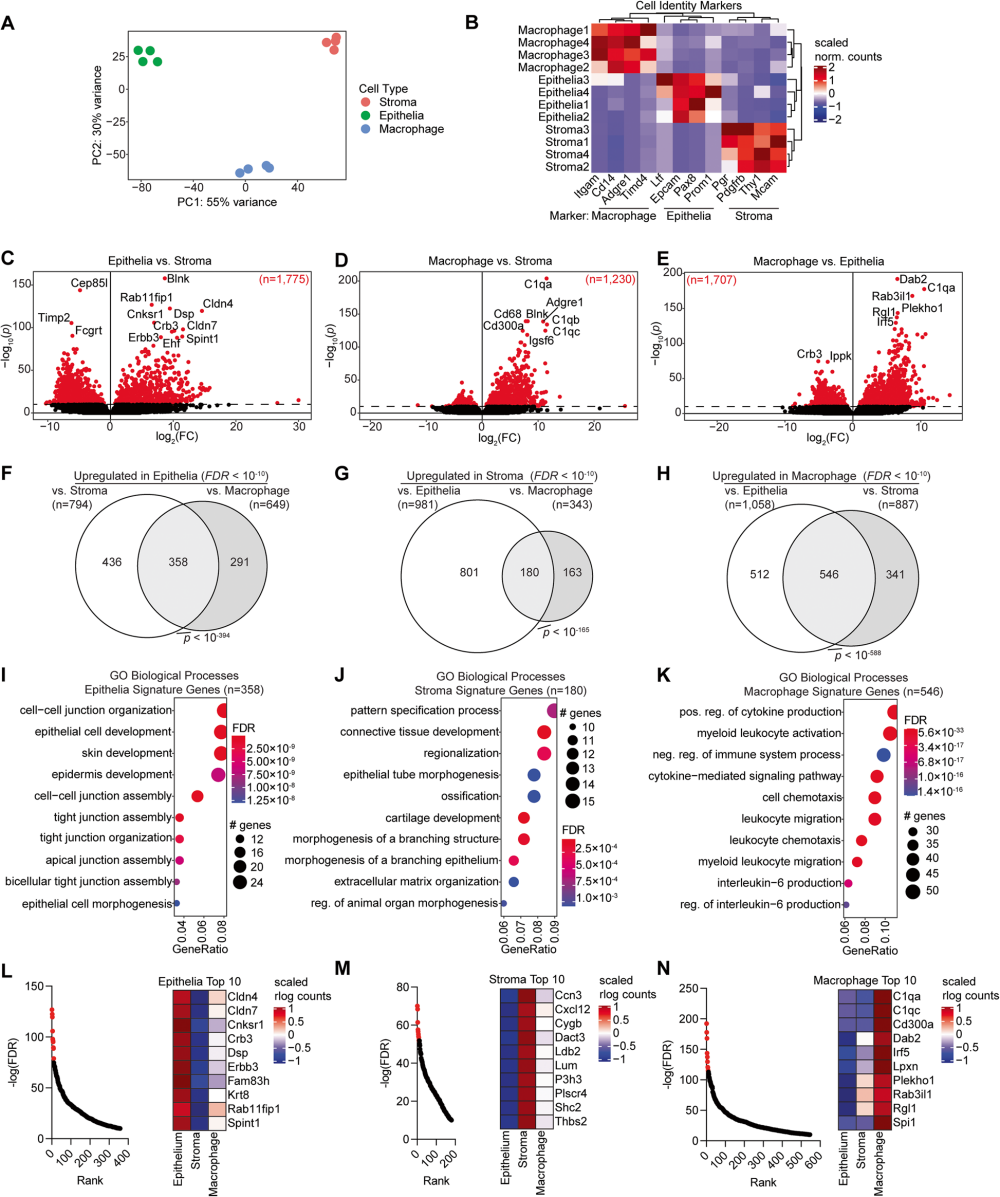
Identification of new markers for endometrial epithelia, stroma, and macrophages by RNA-seq analysis. **A** Principal component analysis of gene expression data from sorted endometrial epithelia, stroma, and macrophages (control diet condition) (*n* = 4 per cell type). **B** Heatmap of gene expression for known macrophage, epithelia, and stroma cell identity markers. **C**-**E** Volcano plot of differentially expressed genes between two cell types. Significantly differentially expressed genes are marked in red (*FDR* < 0.05). **F**–**H** Identification of signature genes for each cell type based on differentially expressed genes against other two cell types as in panels **C**-**E**. Plots are proportional Euler diagrams. Statistic is hypergeometric enrichment. **I**-**K** Enrichment analysis for Gene Ontology (GO) biological processes for signature gene sets identified as in panels **F**–**H**. **L**-**N** Left, ranking of most significantly upregulated genes with respect to epithelia vs. stroma (**L**), stroma vs. epithelia (**M**) and macrophage vs. epithelia (**N**), with top ten genes highlighted in red. Right, heatmap of ten most upregulated genes in given cell type and expression in all three cell types

We first compared the three cell types from the control diet-treated mice to validate our study and identify new marker genes for these cell populations. Epithelia, stroma, and macrophage populations were highly unique from one another based on principal component analysis (Fig. 3A), and cell identity of these populations was confirmed by unique expression of canonical marker genes (Fig. 3B). We identified genes with differential gene expression between the three cell types and found between 1,230 and 1,775 differential genes between each comparison (*FDR* < 10^–10^) (Fig. 3C-E). Utilizing the three comparison sets, we identified 358 signature epithelia genes, 180 signature stroma genes and 546 signature macrophage genes (Fig. 3F-H). We performed enrichment tests for the Gene Ontology (GO) Biological Processes on each of these gene sets and found that the epithelia signature genes were enriched for pathways related to cell–cell junction, tight junction, and epithelial cell development (Fig. 3I) The stroma gene signature was enriched for pathways related to connective tissue development, morphogenesis, and extracellular matrix organization (Fig. 3J). The macrophage gene signature was enriched for pathways related to immune signaling and chemotaxis (Fig. 3K). We used this data to identify the genes most significantly expressed in each cell type by ranking the genes in each signature based on significance (Fig. 3L-N). The most significantly expressed genes in the epithelia were Claudins 4 and 7 (*Cldn4* and *Cldn7*), two integral membrane proteins that determine tight junction permeability [42]. In the endometrial stroma, the most significantly expressed gene was *Ccn3*, an extracellular matrix protein which regulates growth and differentiation [43]. In the endometrial macrophages, the most significantly expressed genes were *C1qa* and *C1qc*, components of the C1q complex of the complement system which can also act as a tumor-promoting factor [44]. These data validate the unique cell populations isolated as epithelia, stroma and macrophage and suggest new marker genes for these cell types.

### Endometrial epithelia of obese mice recapitulate the inflammatory phenotype of tumor models

**Fig. 4:**
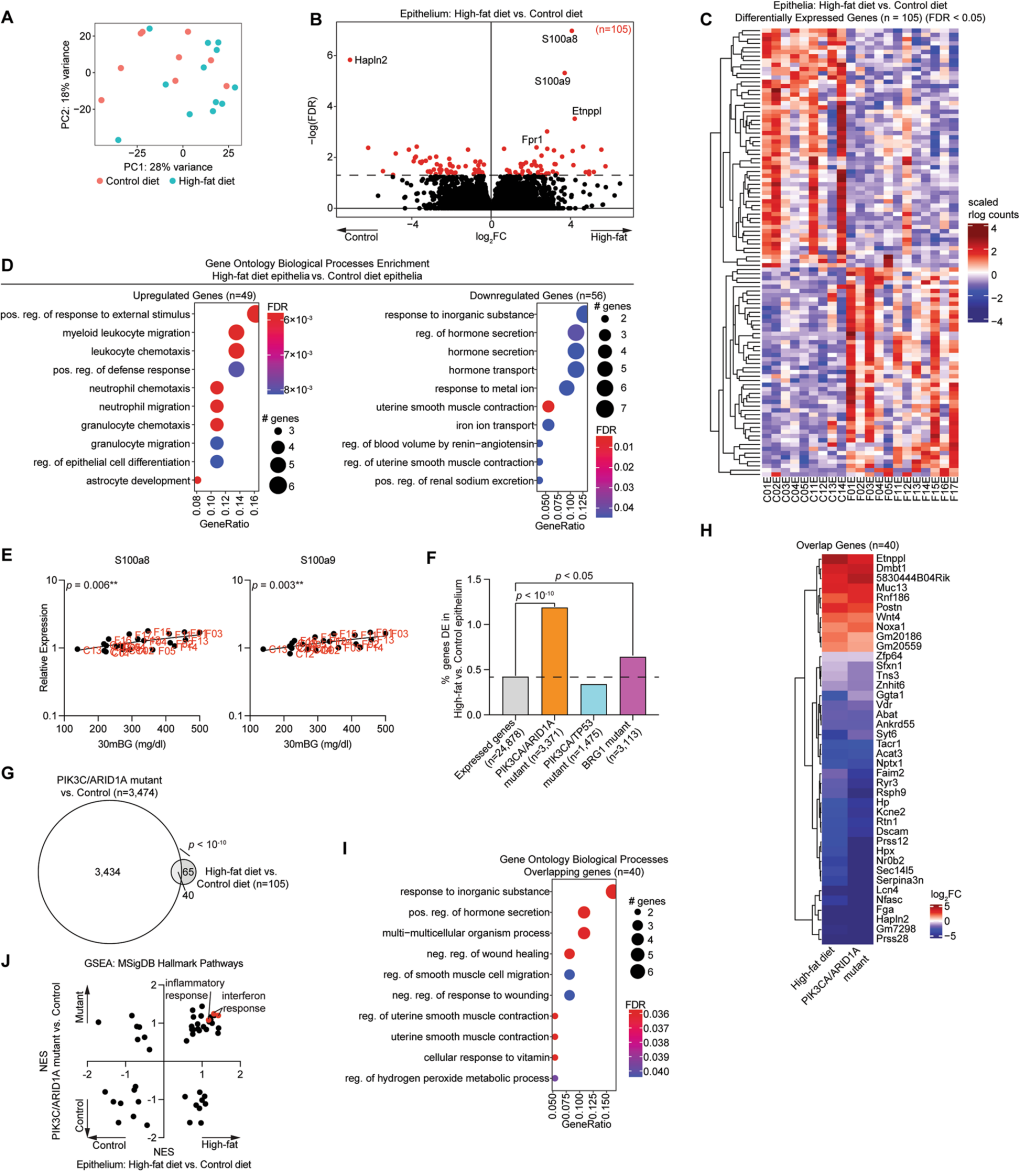
Differential transcriptome analysis of obese endometrial epithelia. **A** Principal component analysis of gene expression data of sorted endometrial epithelia from control diet and high-fat diet. **B** Volcano plot of expressed genes. Significant differentially expressed genes (*FDR* < 0.05) among epithelia are highlighted in red. Genes with *FDR* < 0.001 are denoted with gene symbols. **C** Heatmap of 105 significant differentially expressed genes in all samples. **D** Enrichment analysis for GO biological processes for upregulated genes (*n* = 49) (left) or downregulated genes (*n* = 56) (right). **E** Correlation between glucose tolerance (measurement at 30 min) and expression of *S100a8* (left) and *S100a9* (right). Statistic is Pearson’s correlation. **F** Enrichment of differentially expressed genes in high-fat epithelia for differentially expressed genes in PIK3CA/ARID1A mutant (orange), PIK3CA/TP53 mutant (blue) or BRG1 mutant (purple) epithelia from previously published studies. Statistic is hypergeometric enrichment. **G** Proportional Euler diagram of overlap between differentially expressed genes in high-fat diet vs. control diet epithelia (*n* = 105) and PIK3CA/ARID1A mutant vs. control epithelia (*n* = 3,371). Statistic is hypergeometric enrichment. **H** Heatmap of fold change values for overlapping genes from panel G. **I** Enrichment analysis for GO biological processes with overlapping genes from panel G. **J** Broad GSEA results for high-fat diet vs. control diet epithelia (x-axis) and PIK3CA/ARID1A mutant vs. control epithelia (y-axis). Pathways of interest are highlighted in red

Comparing the control and high-fat diet samples within each cell type, we observed over 100 differentially expressed genes in the epithelia and the stroma (Supplemental Fig. 3A). However, among macrophages, we observed significant differential expression of only a single gene, Gm11814, suggesting an increase in recruitment of macrophages to the uterus without a major impact on their transcriptomic profile (Supplemental Fig. 3B,C). A high amount of variability was observed among the epithelial cell isolates by principal component analysis (Fig. 4A), but we still observed 105 differentially expressed genes (*FDR* < 0.05) (Fig. 4B). Differentially expressed genes between high-fat diet and control diet groups were unique from those observed by comparing mice of varying estrous stage, suggesting an independent effect driven by diet (Supplemental Fig. 4). Among the 105 significant differentially expressed genes, 49 were upregulated and 56 were downregulated in high-fat diet mice relative to the control diet mice (Fig. 4C). Upregulated genes were enriched for GO Biological Processes related to leukocyte migration, while downregulated genes were enriched for GO Biological Processes related to hormone secretion and ion transport (Fig. 4D). Two of the most significantly upregulated genes, *S100a8* and *S100a9*, form the heterodimer complex Calprotectin, which binds calcium and regulates inflammation [45]. We found that the expression of these two genes in the endometrial epithelia is significantly correlated with glucose intolerance (Fig. 4E).

To determine whether the high-fat diet epithelia had transcriptomic changes similar to mice with a uterine pathology, we utilized our previously published RNA-seq datasets from the endometrial epithelia of several genetically engineered mouse models (GEMM) we have developed on a CD-1/B6 outbred background. *LtfCre*^*0/*+^; (Gt)R26Pik3ca^*H1047R^; *Arid1a*^*fl/fl*^ mice (henceforth, PIK3CA/ARID1A mutant mice) develop endometrial hyperplasia and myometrial invasion with features of endometrioid carcinoma, which can also develop an endometriosis phenotype following uterotubal incision surgery [20, 46]. *LtfCre*^*0/*+^; (Gt)R26Pik3ca^*H1047R^*; Trp53*^*fl/fl*^ mice (henceforth, PIK3CA/TP53 mutant mice) develop features of non-invasive hyperplasia, adenocarcinoma, and endometrial intraepithelial carcinoma (the precursor of serous endometrial carcinoma) [31]. *LtfCre*^*0/*+^*; Brg1*^*fl/fl*^ (BRG1 mutant mice) develop an adenomyosis phenotype [30]. The 105 genes differentially expressed in the endometrial epithelia of obese mice were significantly enriched for the differential genes of both the PIK3CA/ARID1A mutant mice and BRG1 mutant mice, without a significant enrichment for the differential genes of PIK3CA/TP53 mutant mice (Fig. 4F), suggesting more similarity to endometrioid carcinoma and benign disease than to serous carcinoma. 38% of genes affected by high-fat diet were also dysregulated in PIK3CA/ARID1A mice (Fig. 4G). In every instance, the direction of differential gene expression of the 40 overlapping genes was the same between obese mice and PIK3CA/ARID1A mutant mice (Fig. 4H). The overlapping set of 40 genes was enriched for GO Biological Processes related to wound healing and muscle contraction (Fig. 4I). Next, we performed Broad Gene Set Enrichment Analysis (GSEA) [38] for the MSigDB Hallmark pathways [47] and compared normalized enrichment score (NES) values for each pathway between models. We observed that the direction of change was consistent for most pathways, with interferon response and inflammatory response pathways positively enriched following either PIK3CA/ARID1A mutation or high-fat diet (Fig. 4J).

### The obese endometrial stroma displays dysregulation of endometrial cancer genes

**Fig. 5:**
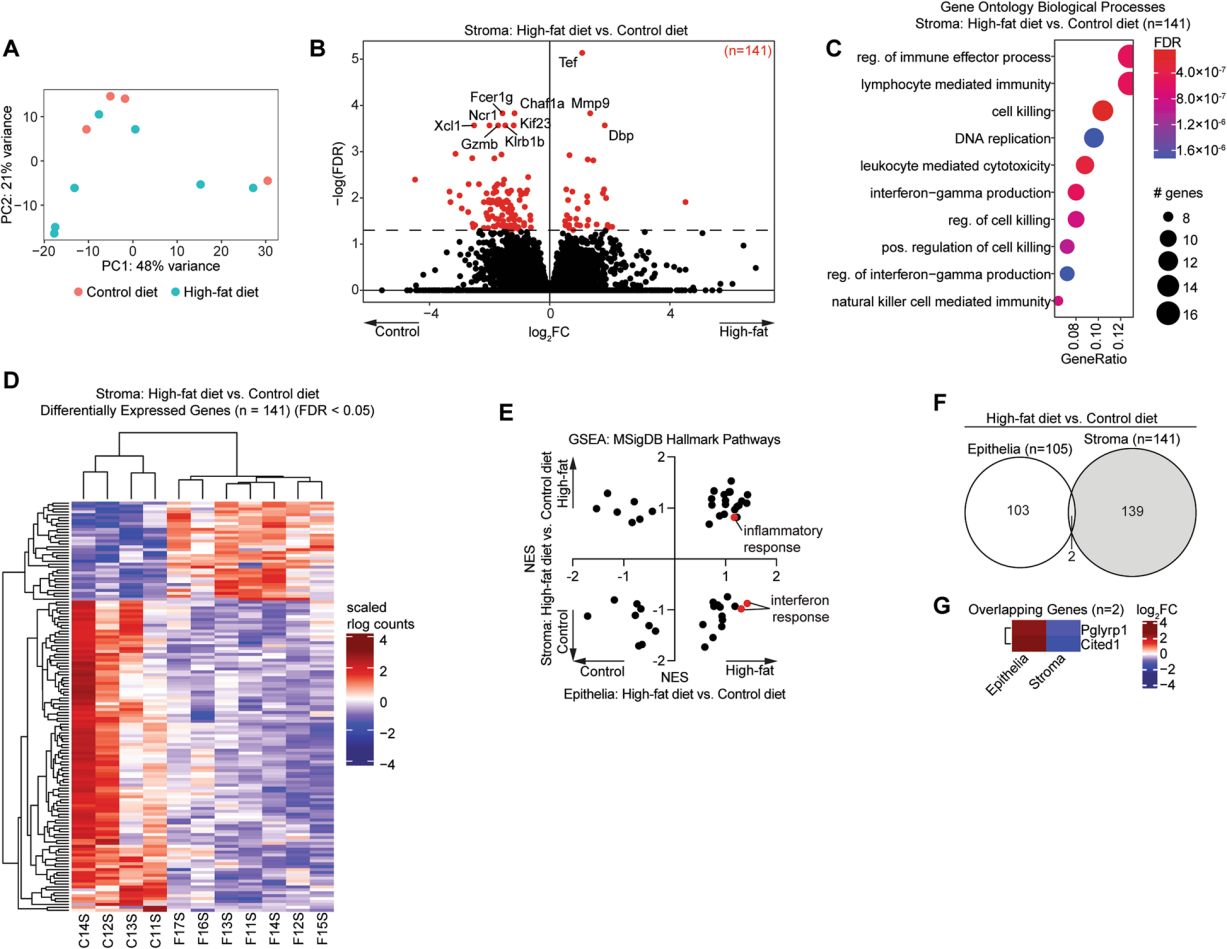
Differential transcriptome analysis of obese endometrial stroma. **A** Principal component analysis of gene expression data from sorted endometrial stroma of control diet and high-fat diet. **B** Volcano plot of expressed genes. Significant differentially expressed genes (*FDR* < 0.05) among stroma are highlighted in red. Genes with *FDR* < 0.001 are denoted with gene symbols. **C** Enrichment analysis for GO biological processes for differentially expressed genes (*n* = 141). **D** Hierarchical clustering of 141 significant differentially expressed genes in all samples. **E** Broad GSEA results for high-fat diet vs. control diet epithelia (x-axis) and stroma (y-axis). Noteworthy pathways are highlighted in red. **F** Proportional Euler diagram of overlap between differentially expressed genes in high-fat diet vs. control diet epithelia (*n* = 105) and stroma (*n* = 141). Statistic is hypergeometric enrichment. **G** Heatmap of fold change values for overlapping genes from panel F

Next, we investigated the effects of obesity on the endometrial stroma. As with the endometrial epithelia, we observed variance between stromal samples based on principle component analysis (Fig. 5A). We identified 141 differentially expressed genes in the endometrial stroma of obese mice compared with control mice (Fig. 5B). These genes had no significant overlap with genes affected by estrous cycle stage in the stroma (Supplemental Fig. 4). The 141 genes were enriched for GO Biological Processes related to immune processes and cell killing (Fig. 5C). Although there was variability between samples, samples clustered together based on their experimental condition following hierarchical clustering of the 141 genes (Fig. 5D). We performed Broad GSEA for the MSigDB Hallmark pathways and observed many differences between the response of endometrial epithelia and endometrial stroma to obesity. The inflammatory response pathway was upregulated in both the epithelia and the stroma, though the interferon response pathways were downregulated in the endometrial stroma but upregulated in the endometrial epithelia, suggesting a unique role for the endometrium in promoting innate immunity during obesity (Fig. 5E). Obesity-induced gene expression changes in the epithelia vs. stroma were almost entirely distinct, indicating cell type-specific changes to the transcriptome. Although the number of individual genes with differential gene expression in both the epithelia and the stroma was not significant, we did identify two genes that were upregulated in the epithelia and downregulated in the stroma (Fig. 5F). Confirming the differences in innate immunity between the two cell types in response to obesity, we observed that *Pglyrp1* was upregulated in the epithelia and downregulated in the stroma (Fig. 5G).

### The uterine peripheral clock is impacted by obesity

**Fig. 6:**
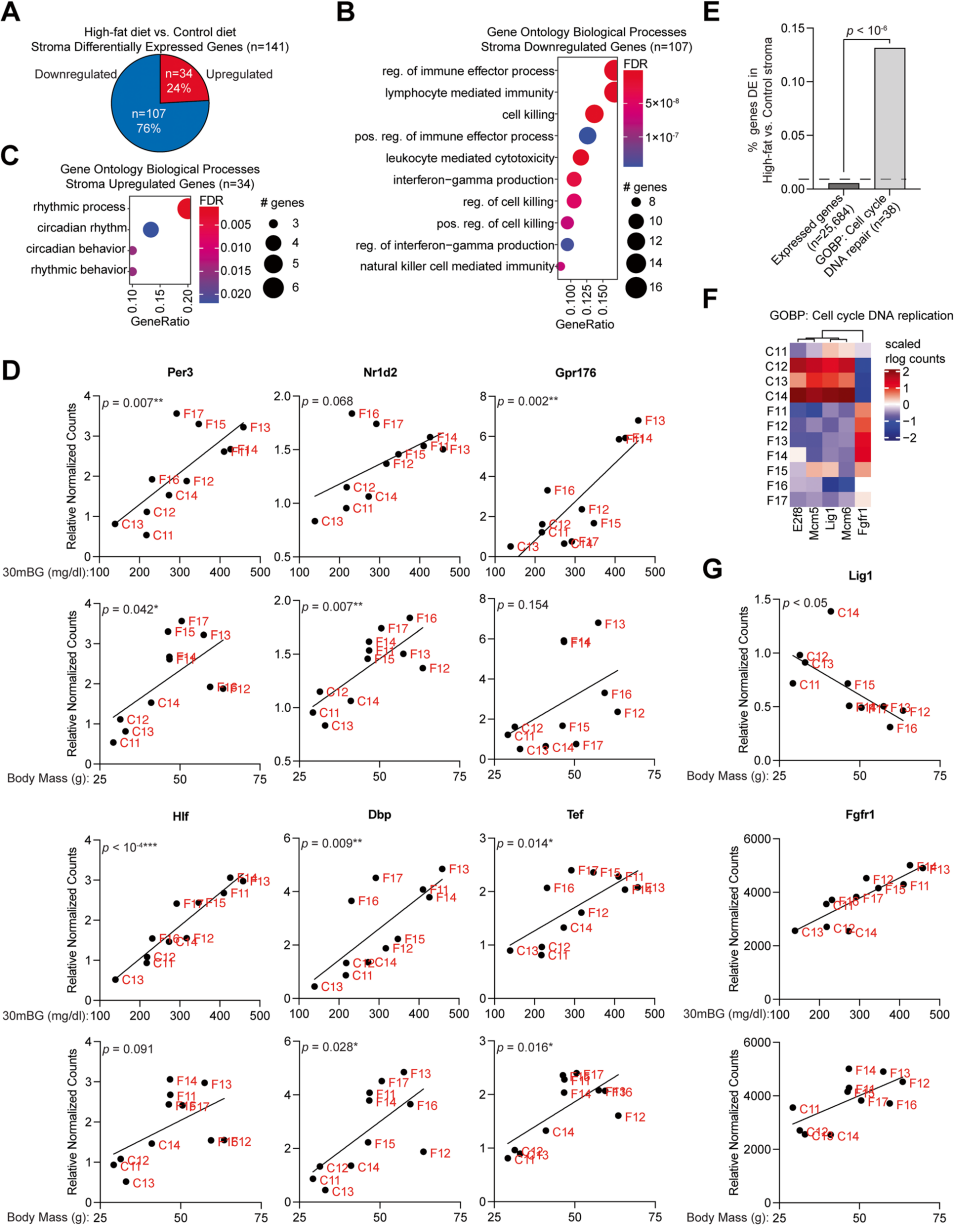
Alterations to the circadian clock in obese endometrial stroma. **A** Distribution of genes which are significantly upregulated vs. downregulated in high-fat stroma. **B**-**C** Enrichment analysis for GO biological processes for downregulated genes (*n* = 107) (**B**) or upregulated genes (*n* = 34) (**C**). **D** Correlation between glucose tolerance (measurement at 30 min) or body mass and expression of rhythmic process genes *Per*, *Nr1d2*, *Gpr176*, *Hlf*, *Dbp*, and *Tef*. Statistic is Pearson’s correlation. **E** Enrichment analysis for GO biological process Cell Cycle DNA Repair. Statistic is hypergeometric enrichment. **F** Heatmap of altered genes (*n* = 5) from Cell Cycle DNA Repair pathway. **G** Correlation between body mass and *Lig1* (top) or *Fgfr1* (bottom) expression or glucose tolerance (measurement at 30 min) and *Fgfr1* expression (middle). Statistic is Pearson’s correlation

In the stroma, most genes impacted by high-fat diet (76%) were downregulated (Fig. 6A). Downregulated genes (*n* = 107) were enriched for GO Biological Processes related to immune processes and cell killing (Fig. 6B), similar to the total set of DE genes (compared with Fig. 5C). Among upregulated genes (*n* = 34), all enriched GO Biological Processes were related to circadian rhythm (Fig. 6C). For the six genes found within the rhythmic process pathway, stromal gene expression was significantly correlated with body mass of the mouse (*Nr1d2*), glucose intolerance (*Gpr176*, *Hlf*) or both (*Per3*, *Dbp*, *Tef*) (Fig. 6D), suggesting that obesity can impact the peripheral clock of the uterus (Fig. 6D). Circadian clock genes can also regulate the cell cycle [48], so we explored whether cell cycle may be affected in the high-fat diet stroma. The GO Biological Process of “cell cycle DNA repair” was strongly enriched among differentially expressed genes (Fig. 6E). Of the affected genes, most were downregulated in the high-fat diet stroma (Fig. 6F). *Lig1* (DNA ligase 1) expression was negatively correlated with body mass, while *Fgfr1* expression was positively correlated with both glucose intolerance and body mass (Fig. 6G).

### Diverse response to high-fat diet leads to differential epithelial and macrophage gene programs

**Fig. 7:**
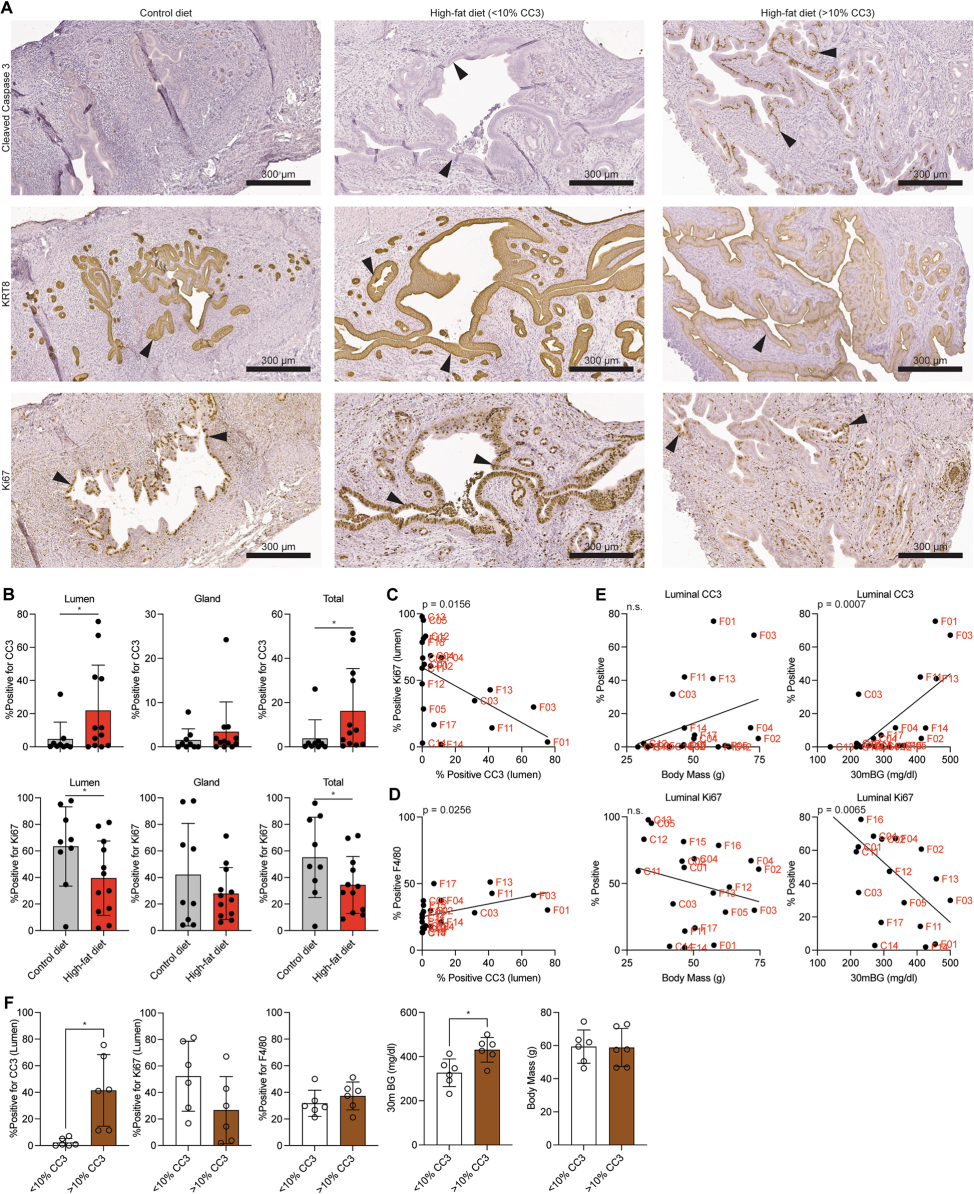
Differential apoptosis among high-fat endometrial epithelia. **A** Immunohistochemistry for Ki67 (top row, indicates proliferation), Cleaved Caspase 3 (CC3) (middle row, indicates apoptosis) and KRT8 (bottom row, endometrial epithelia marker) in uteri from mice on control diet (left column) or high-fat diet (middle and right columns). Two samples are shown for high-fat diet mice, one without luminal CC3 staining (middle) and one with high CC3 staining (right). Arrowheads indicate positive staining in endometrial epithelia. **B** Quantification of CC3 (top row) and Ki67 (bottom row) among luminal (left column), glandular (middle column) or total (right column) endometrial epithelia. Statistic is ANOVA. **C** Correlation between luminal CC3 and Ki67 among all mice. Statistic is Pearson’s correlation. **D** Correlation between luminal CC3 and endometrial F4/80 among all mice. Statistic is Pearson’s correlation. **E** Correlations between luminal CC3 (top row) or Ki67 (bottom row) and body mass in grams (left column) or glucose tolerance (measurement at 30 min, mg/dl) (right column) among all mice. Statistic is Pearson’s correlation. **F** Histograms comparing luminal CC3, luminal Ki67, endometrial F4/80, glucose tolerance (measurement at 30 min, mg/dl) and body mass (g) between high-fat diet mice with < 10% luminal CC3 or > 10% luminal CC3. Statistic is ANOVA. *p < 0.05, n.s. is non-significant

We next explored whether obesity led to differences in proliferation and apoptosis in the mouse uterus. We observed a significant increase in Cleaved Caspase 3 (CC3) in the luminal, but not glandular, epithelial layers of the endometrium in some, but not all, of the high-fat diet mice (Fig. 7A, B). The epithelium was identified by KRT8 staining (Fig. 7 A,B), and compared to negative controls (Supplemental Fig. 5). Conversely, we observed a specific decrease in Ki67 in the luminal epithelial layer (Fig. 7A,B). Luminal CC3 was inversely correlated with luminal Ki67 (Fig. 7C), indicating a less proliferative and more apoptotic epithelial lumen. Luminal CC3 was also positively correlated with the F4/80 macrophage marker (Fig. 7D). Changes in luminal CC3 and Ki67 positive staining patterns correlated with glucose intolerance (Fig. 7E), indicating that glucose intolerance, apoptotic endometrial lumen and macrophage abundance are correlated features (Fig. 7E). Staining for CC3, Ki67 and F4/80 was not impacted by estrous stage (Supplemental Fig. 4K-M).

**Fig. 8:**
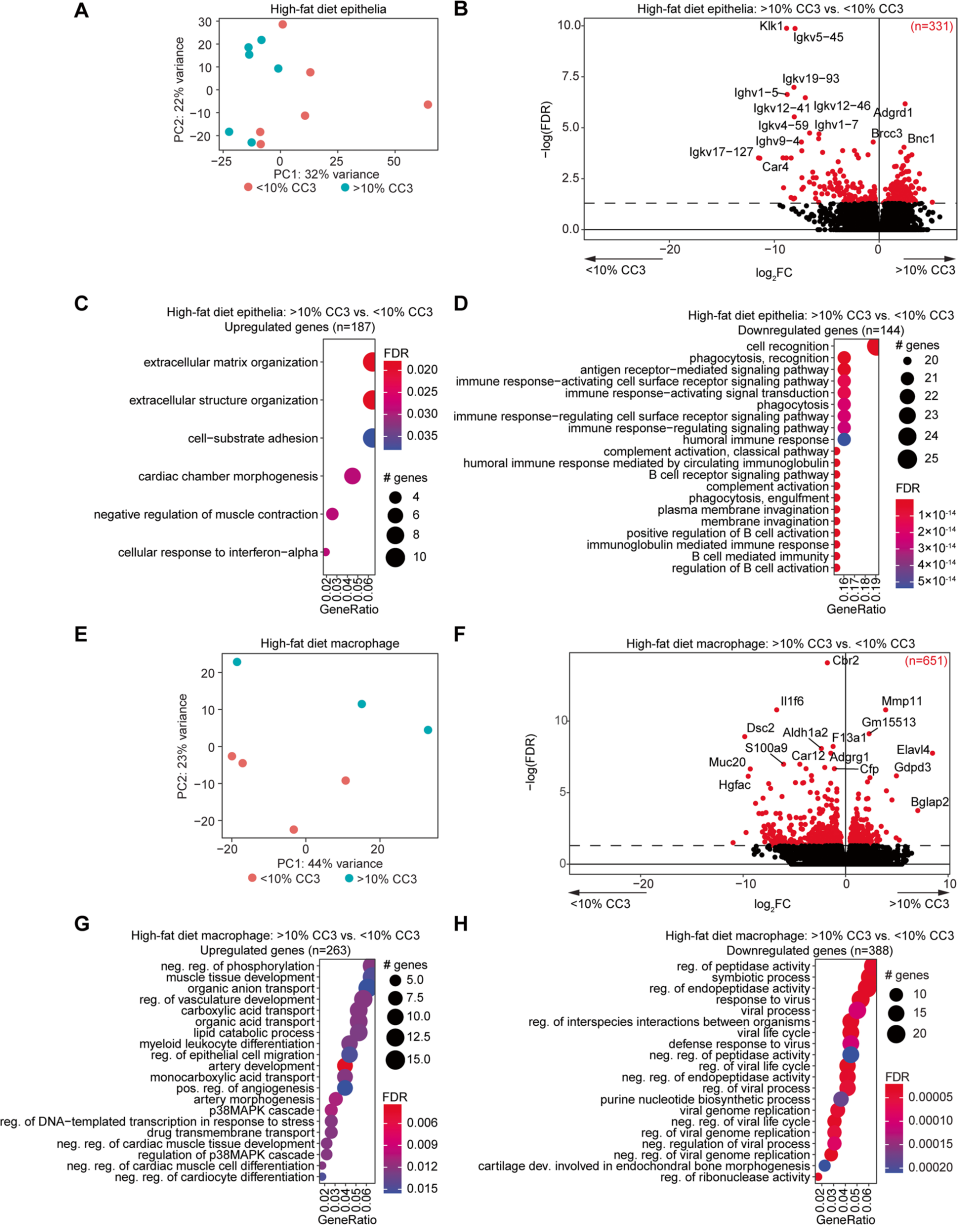
Differential gene expression profiles among mice with apoptotic luminal epithelia. **A** Principal component analysis of gene expression data from high-fat diet endometrial epithelia based on luminal CC3, > 10% or < 10%. **B** Volcano plot of expressed genes in epithelia of two groups. Significant differentially expressed genes (*FDR* < 0.05) among > 10% CC3 epithelia compared to < 10% CC3 epithelia are highlighted in red. Genes with *FDR* < 0.001 are denoted with gene symbols. **C**, **D** Enrichment analysis for GO biological processes for upregulated genes (*n* = 187) (C) or downregulated genes (*n* = 144) (**D**). **E** Principal component analysis of gene expression data from high-fat diet macrophages based on luminal CC3. **F** Volcano plot of expressed genes in macrophages of two groups. Significant differentially expressed genes (*FDR* < 0.05) are highlighted in red. Genes with *FDR* < 0.001 are denoted with gene symbols. **G**, **H** Enrichment analysis for GO biological processes for upregulated genes (*n* = 263) (**G**) or downregulated genes (*n* = 388) (**H**) among macrophages from > 10% CC3 and < 10% CC3 groups

Observing the range in glucose intolerance, epithelial apoptosis, and macrophage abundance among the high-fat diet mice, we next considered the high-fat diet mice as two groups: those with > 10% CC3 positive staining in the epithelial lumen, and those with < 10% CC3 positive staining. High-fat diet mice with > 10% CC3 also displayed greater glucose intolerance, without significant differences in luminal Ki67, endometrial F4/80 or body mass (Fig. 7F). Exploring the differential gene expression between the epithelia of mice with < 10% CC3 or > 10% CC3, we observed differences by principal component analysis (Fig. 8A, compared to Fig. 4A). Consistent with this, there were 331 genes differentially expressed in comparisons between the epithelia of high-fat diet mice grouped by > 10% CC3 versus < 10% CC3 positive staining (Fig. 8B). Genes upregulated in the > 10% CC3 group were enriched for pathways related to extracellular matrix organization (Fig. 8C), while downregulated genes were enriched for pathways related to cell recognition and immune-response activation (Fig. 8D). Genes differentially expressed in the > 10% CC3 group were significantly overlapped with genes differentially expressed in mice in the estrus stage compared to other stages, with 19% of genes being shared between these analysis (Supplemental Fig. 4). However, the majority were unique to the > 10% CC3 vs. < 10% CC3 analysis.

Macrophages from each of the > 10% CC3 versus < 10% CC3 groups were also separated based on principal components (Fig. 8E, compared to Supplemental Fig. 3B), and 651 differentially expressed genes were observed between the two groups (Fig. 8F). A broad spectrum of pathways were enriched in the genes upregulated by the macrophages within the > 10% CC3 group (Fig. 8G), while downregulated genes were related to viral response (Fig. 8H). Genes in this group were not impacted by estrous stage in the macrophages (Supplemental Fig. 4).

**Fig. 9:**
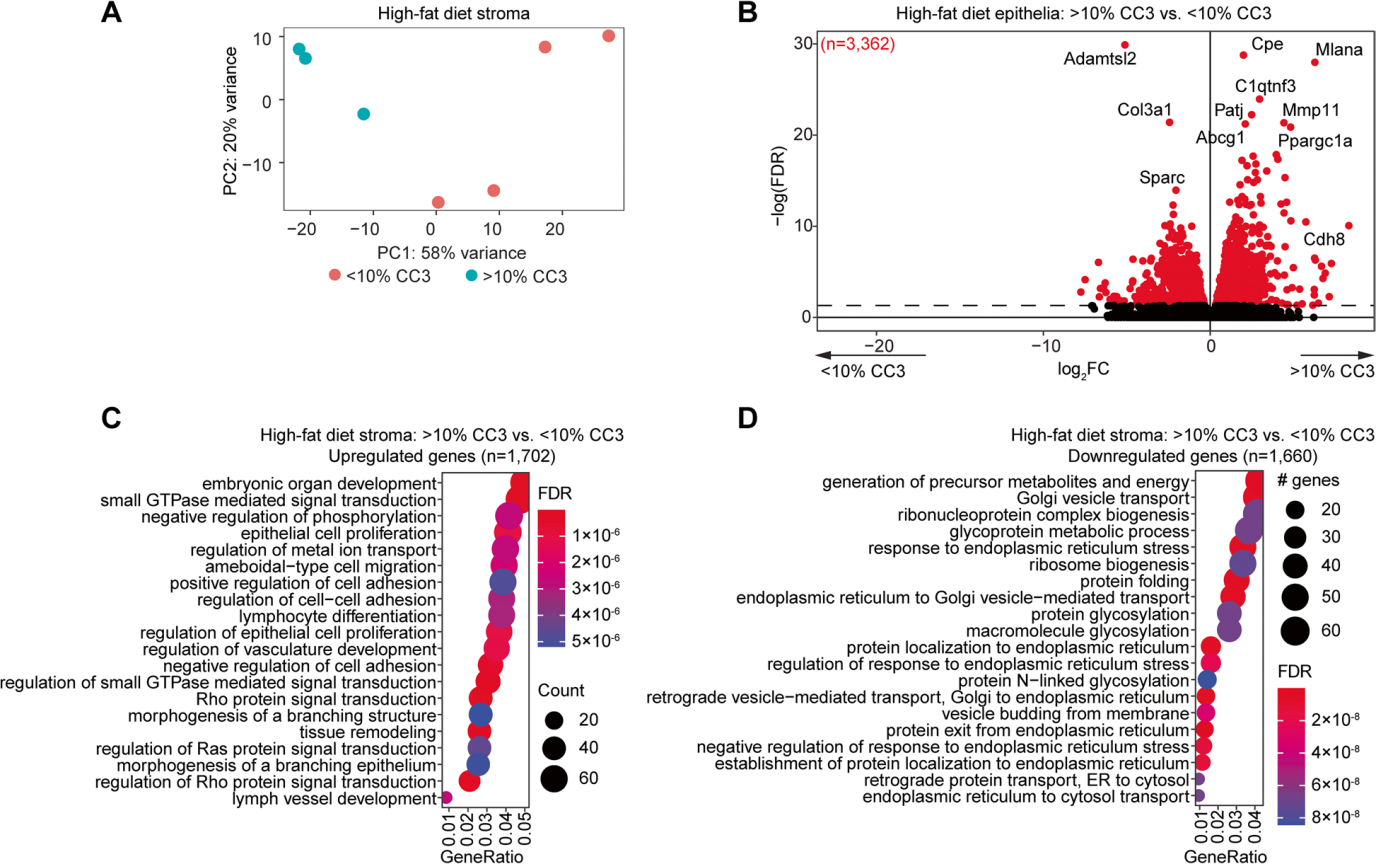
Differential stroma expression profiles among mice with apoptotic luminal epithelia. **A** Principal component analysis of gene expression data from high-fat diet endometrial stroma based on luminal epithelia CC3, > 10% or < 10%. **B** Volcano plot of expressed genes in stroma of two groups. Significant differentially expressed genes (*FDR* < 0.05) among > 10% CC3 epithelia compared to < 10% CC3 epithelia are highlighted in red. Genes with *FDR* < 0.001 are denoted with gene symbols. **C**,**D** Enrichment analysis for GO biological processes for upregulated genes (*n* = 1,702) (**C**) or downregulated genes (*n* = 1,660) (**D**)

Stroma from each group were also clearly separated by gene expression profiles (Fig. 9A), with 3,361 genes differentially expressed between the two groups. Upregulated genes were enriched for pathways related to organ development, cell adhesion and regulation of epithelia (Fig. 9C), while downregulated genes were enriched for pathways related to golgi and endoplasmic reticulum (Fig. 9D). These genes were unique from those impacted by estrous stage in the stroma (Supplemental Fig. 4).

## Discussion

The global incidence of obesity has increased rapidly over the past 50 years, reaching pandemic levels [49]. Among the numerous health problems associated with obesity, a significant number of cancer deaths result from obesity [50]. Particularly, the incidence of EC is rising steadily due to the increased incidence of obesity [10, 12, 13]. A wide variety of metabolic conditions are linked to both obesity and EC, including hyperinsulinemia, metabolic syndrome, and type 2 diabetes [10, 13]. Metabolic risk factors also contribute to idiopathic infertility [3]. In this study, we measured the impact of obesity on three different cell types in the uterus, endometrial epithelia, stroma and macrophages, by performing gene expression and pathological analyses. High-fat diet induced unique differential gene expression in the epithelia and stroma compared to control diet, with a minimal effect on uterine macrophages. The differential effects within cell types validates our model, in that different cell types should respond differently to the systemic phenotype of obesity, as has been reported in a direct comparison of adipose and skeletal muscle [51].

S100A8 and S100A9 subunits form the calprotectin complex, a secreted factor that utilizes calcium as a cofactor to chelate zinc as part of the innate immune system [52]. Chelation of zinc can affect cell migration and extracellular matrix properties through inhibition of matrix metalloproteases. Calprotectin also stimulates inflammation through its activities as a cytokine [45] and stimulates leukocyte migration and adhesion [53]. Measurements of fecal calprotectin can be used as a marker for gastrointestinal inflammation [54]. Bacterial infection has been shown to increase the abundance of S100A9 in the bovine uterine proteome [55]. The expression of S100A8 and S100A9 is increased in the endometrial decidua tissue of women with recurrent early pregnancy loss [56]. Upregulation of calprotectin in the obese endometrial epithelia may contribute to the decreased endometrial decidualization observed in mouse and human cell line models [57]. Pglyrp1, found to be upregulated in the epithelia and downregulated in the stroma (Peptidoglycan recognition protein 1, also called Tag7) is a mediator of innate immunity which can also interact with calcium binding protein S100A4 [58]. In addition to calprotectin, we also report a downregulation of endoplasmic reticulum calcium transporter Ryr3 in the obese endometrial epithelia (Fig. 4H), suggesting that obesity-induced disruption of calcium signaling may impact innate immune processes in this cell type.

The uterus of non-pregnant mice and rats possesses a functional molecular clock, while pregnancy prolongs the uterine circadian rhythm [59, 60]. Mice with a dominant negative *Clock* gene, *ClockΔ19,* are unsuccessful at parturition, likely due to loss of periodic circadian rhythm gene expression in the uterus [61, 62]. Previously, the periodic expression of *Per3*, *Tef* and *Dbp* were shown to be regulated in stromal cells of pregnant rats [63]. In our study, these three genes were differentially expressed in obese mice, and gene expression was significantly correlated with decreased glucose tolerance. These findings suggest that the circadian clock of the endometrial stroma becomes dysregulated during obesity, a phenomenon which may influence the uterine microenvironment and impact fertility and cancer pathogenesis. Much of the previous work in the circadian rhythm field has focused on the effects on estrogen signaling in the uterus. Uterine explants from ovariectomized rats display a reduced period of expression for *Per2* following treatment with estrogen [64]. Neurons of the suprachiasmatic nucleus regulate the estrous cycle through expression of vasoactive intestinal peptide, and genetic loss of vasoactive intestinal peptide in female mice decrease reproductive success because of disrupted estrogen signaling [65]. However, peripheral clocks are highly regulated by several hormones, including melatonin, which is reduced endogenously with obesity [66]. Melatonin is primarily produced by the pineal gland and acts through its two G-protein coupled receptors in target tissues, including the pancreas, in which it can regulate insulin production [66]. Furthermore, melatonin can reduce intrauterine inflammation and prevent preterm birth [67]. Additional studies will be needed to characterize the mechanisms by which obesity disrupts the uterine peripheral clock, and the impacts this has on both fertility and disease pathogenesis.

We observed transcriptomic changes in obese mouse epithelia that were comparable to those observed in our endometrial cancer GEMMs. This may reflect similar metabolic conditions among both the obese microenvironment and tumor microenvironment. However, only a fraction of the differentially expressed genes in the endometrial GEMMs were affected by obesity. This is an expected result, as obesity alone is not sufficient for endometrial tumorigenesis [68]. While high-fat diet induced obesity does not appear to be sufficient for endometrial hyperplasia or endometrial tumorigenesis, these results demonstrate a subset of the transcriptomic changes occurring in the obese mouse endometrial epithelium partially reflect those occurring in an endometrial cancer model.

Recently, several studies have identified that somatic driver mutations that are thought to contribute to EC pathogenesis, including *PIK3CA*, *KRAS* and *PIK3R1*, can exist in normal uteri without cancer [69–72]. We have shown that obesity regulates inflammatory pathway genes in the endometrial epithelia and disrupt the peripheral clock in the endometrial stroma. The stroma also showed downregulation of immune-related genes, which could be functionally important, but may also be the result of differences in the profiled cell populations in the impure fraction. Dysregulation of the endometrial stroma can contribute to epithelial phenotypes, as a key function of the endometrial stroma is to regulate the proliferation and differentiation of the epithelia through responses to progesterone and estrogen signaling [73, 74]. We observed cooperative gene expression changes among high-fat diet epithelia with those in a model with features of endometrioid carcinoma, but not intraepithelial carcinoma.

Finally, we observed distinct differences in epithelia, stroma and macrophage gene expression among mice with apoptotic luminal epithelia (> 10% CC3 vs. < 10% CC3). Epithelia downregulated immune-activating cell recognition markers, while macrophages downregulated viral defense responses, suggesting that macrophages may become unresponsive to damaged epithelium in this context, leading to a persistence of apoptotic cells. Stroma of these mice upregulated epithelial cell proliferation genes, suggesting a response to epithelia undergoing apoptosis. Given that endometrial cancers arise from the endometrial epithelia, modifications to stromal regulation of epithelia resulting from obesity could indicate a mechanism by which obesity can create a tumor-prone microenvironment. These results suggest that pathological changes in the endometrium are associated with the metabolic phenotype of obesity, but that changes within each cell compartment are variable among a diverse outbred strain of mice. Further studies will be needed to determine if this aspect of obesity may contribute to endometrial cancer pathogenesis.

## Supplementary Information

**Additional file 1:** **Supplemental Figure 1.** Increased macrophages in the high-fat diet endometrium. **Supplemental Figure 2.** Purification of endometrial cells. **Supplemental Figure 3.** Differential gene expression analysis of high-fat diet uterine macrophages. **Supplemental Figure 4.** Impact of estrous cycle stage on differential gene expression. **Supplemental Figure 5.** Negative immunohistochemistry staining.

**Additional file 2: Supplemental Data: Table 1.** Differential Gene Expression.

## Data Availability

Newly generated RNA-seq data from control diet and high-fat diet epithelia, stroma, and macrophages we deposited at GEO accession series GSE208586. Previously published RNA-seq data was access from GEO accession series GSE129784, GSE152663 and GSE184499.
