# Supplementary material for: Obesity alters the mouse endometrial transcriptome in a cell context-dependent manner
Source: Reprod Biol Endocrinol. 2022 Nov 24;20:163. doi: 10.1186/s12958-022-01030-0 (PMC9686036; doi:10.1186/s12958-022-01030-0)
Supplement: Supplementary file 1 — Additional file 1: Supplemental Figure 1. Increased macrophages in the high-fat diet endometrium. Supplemental Figure 2. Purification of endometrial cells. Supplemental Figure 3. Differential gene expression analysis of high-fat diet uterine macrophages. Supplemental Figure 4. Impact of estrous cycle stage on differential gene expression. Supplemental Figure 5. Negative immunohistochemistry staining. [file 12958_2022_1030_MOESM1_ESM.pdf]

Obesity alters the mouse endometrial transcriptome in a cell context-dependent manner

Wilson et al. 2022

Supplemental Figures

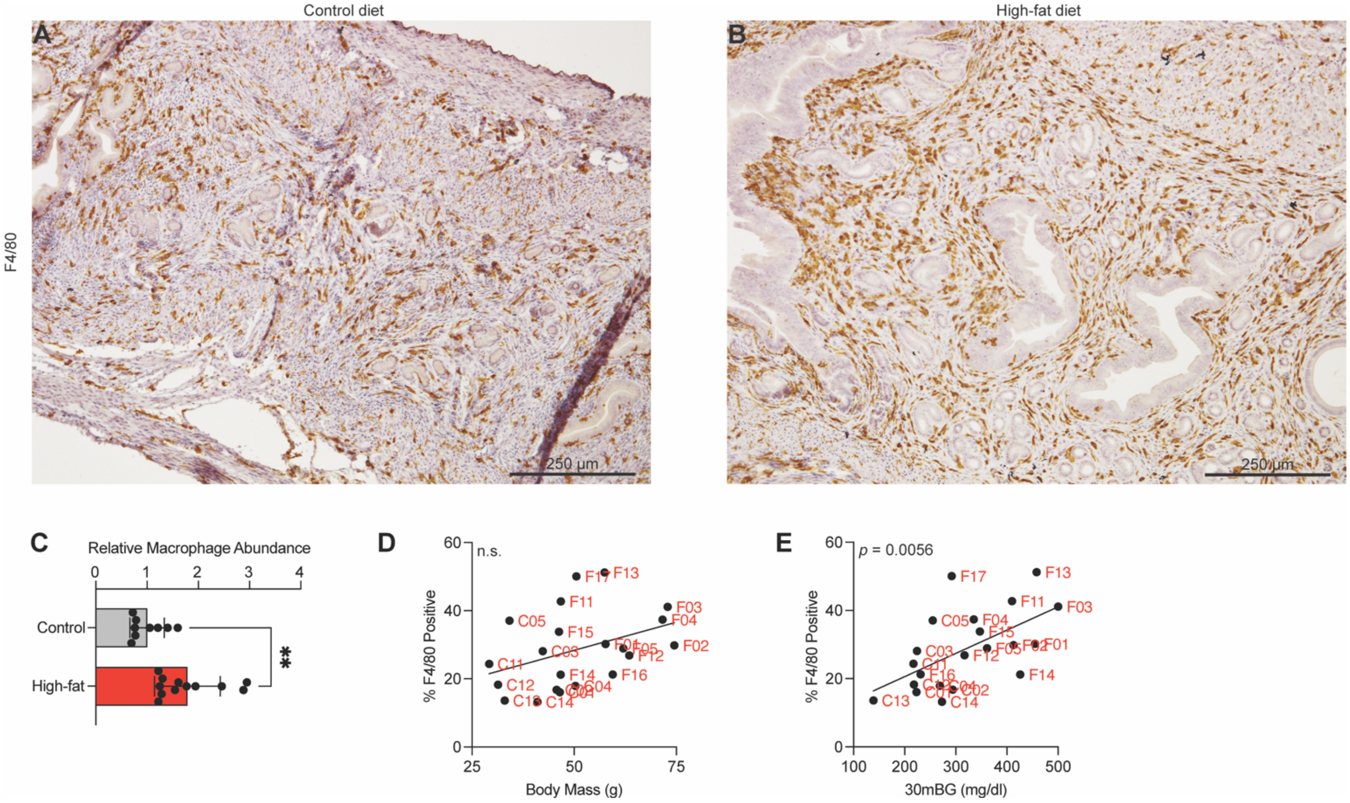

Supplemental Figure 1. Increased macrophages in the high-fat diet endometrium. (A-B) Immunohistochemistry for F4/80 macrophage marker in control diet (A) and high-fat diet (B) endometrium. (C) Relative macrophage abundance as determined by quantitative immunohistochemistry. F4/80+ cells were normalized to total number of cells. (D-E) Correlation between relative abundance of F4/80+ cells and body mass (in grams, panel D) or glucose tolerance (measurement at 30 min, mg/dl, panel E). Statistic is Pearson's correlation.

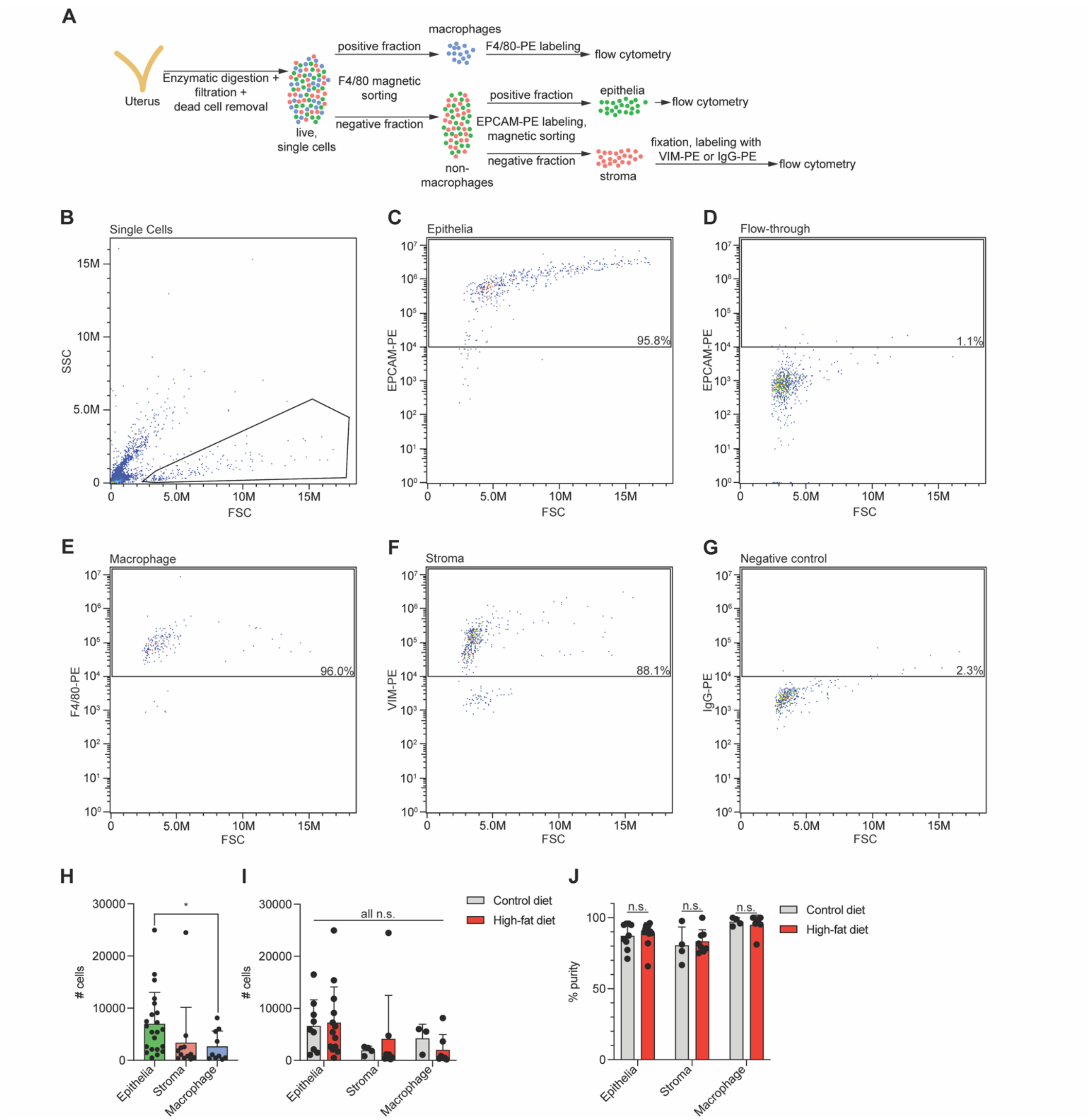

Supplemental Figure 2. Purification of endometrial cells. (A) Outline of magnetic sorting protocol. (B) Flow cytometry gating for single cells. (C-G) Flow cytometry analysis of single cells from positively selected EPCAM-PE+ epithelia (C), negatively-selected epithelia (D), positively selected F4/80-PE+ macrophages (E), fixed VIM-PE+ stroma (F), fixed IgG-PE+ stroma (G). Percentage of cells within population is indicated. (H) number of cells isolated from epithelia (n = 22), stroma (n = 12) or

macrophage (n = 12) samples, mean ± S.D. Statistic is two-tailed, unpaired t-test . (I) Number of cells based on diet condition for epithelia (n = 9 control, n = 13 high-fat), stroma (n = 4 control, n = 8 high-fat), or macrophage (n = 4 control, n = 6 high-fat), mean ± S.D. Statistic is two-tailed, unpaired t-test. (J) Purity of cells based on diet condition for epithelia (n = 9 control, n = 13 high-fat), stroma (n = 4 control, n = 8 high-fat), or macrophage (n = 4 control, n = 6 high-fat), mean ± S.D. Statistic is two-tailed, unpaired Mann-Whitney U. *$p < 0.05$, n.s. is non-significant.

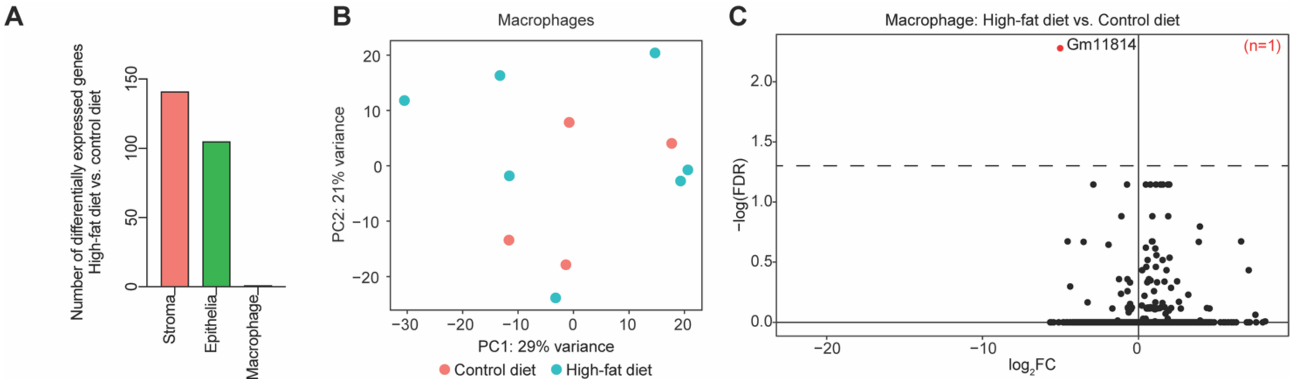

Supplemental Figure 3. Differential gene expression analysis of high-fat diet uterine macrophages. (A) Number of genes differentially expressed in high-fat diet samples compared to control diet samples within stroma, epithelia and macrophage cell types. (B) Principal component analysis of gene expression data from sorted endometrial macrophages from control diet and high-fat diet mice. (C) Volcano plot of expressed genes. Significantly differentially expressed genes (*FDR* < 0.05) among macrophages are highlighted in red. Genes with *FDR* < 0.001 are denoted with gene symbols.

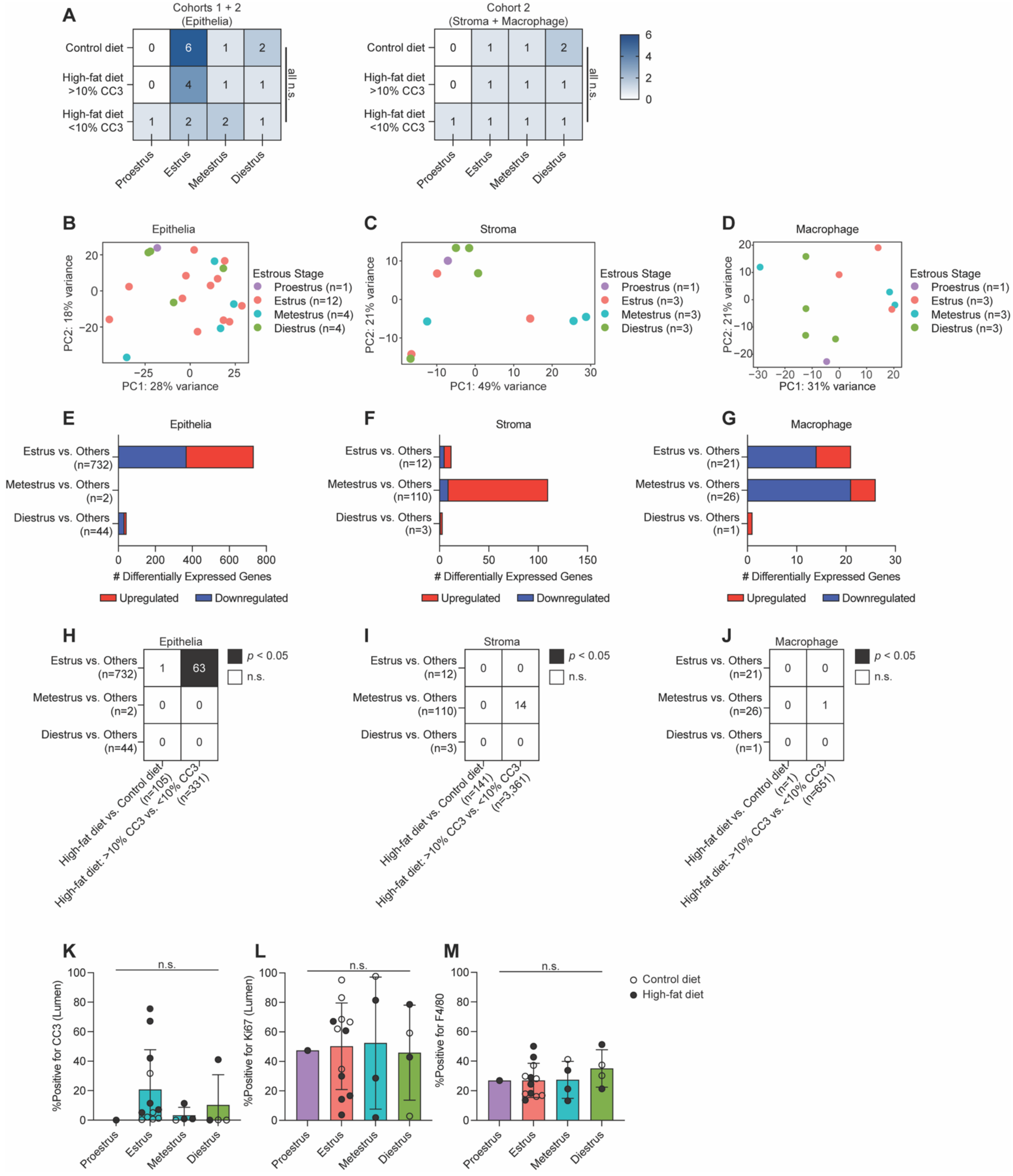

Supplemental Figure 4. Impact of estrous cycle stage on differential gene expression. (A) Heatmap representation of estrous cycle staging by vaginal swab test at time of uteri collection separated into

groups for control diet and high-fat diet, including subgroups for >10% CC3 and <10% CC3 in luminal epithelia (Figure 7), for combined cohorts 1 and 2 (left) or cohort 2 alone (right). Statistic is Chi-square. (B-D) Principal component analysis of gene expression data from epithelia (panel B), stroma (panel C) and macrophage (panel D) based on estrous cycle at time of collection. (E-G) Histograms representing differential gene expression among epithelia (panel E), stroma (panel F) and macrophage (panel G), with the number of upregulated genes shown in red and the number of downregulated genes shown in blue. (H-J) Heatmap representation of hypergeometric enrichment tests for similarity between differential gene expression based on diet or CC3 expression and differential gene expression based on estrous stage among epithelial (panel H), stroma (I) or macrophage (J). (K-M) Histogram representation of luminal CC3 (K), luminal Ki67 (L) and F4/80 immunohistochemical staining based on estrous stage at time of collection. Statistic is ANOVA.

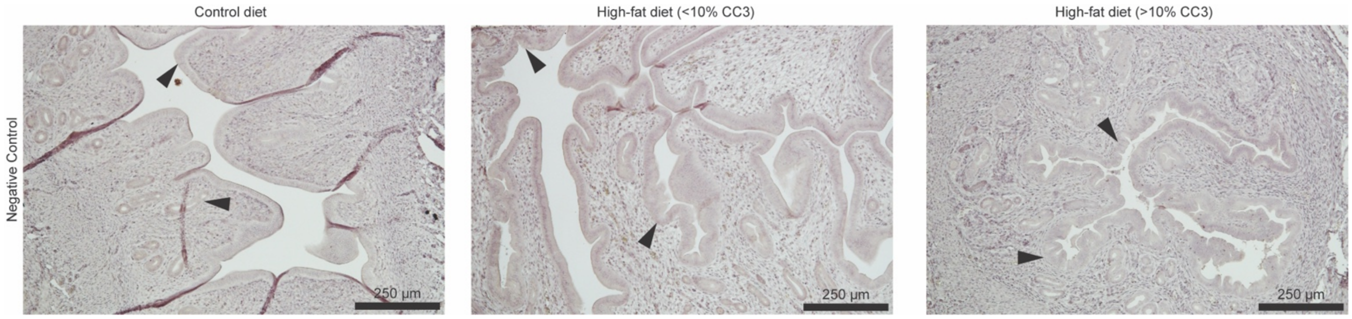

Supplemental Figure 5. Negative immunohistochemistry staining. Negative immunohistochemistry for control and high-fat diet mice. Two samples are shown for high-fat diet mice, one which was without luminal CC3 staining (middle) and one which had high CC3 staining (right), as presented in Figure 7A. Arrowheads indicate endometrial epithelia.
